# Supplementary material for: Effects of Quercetin-Loaded Nanoparticles on MCF-7 Human Breast Cancer Cells
Source: Medicina (Kaunas). 2019 Apr 22;55(4):114. doi: 10.3390/medicina55040114 (PMC6524048; doi:10.3390/medicina55040114)
Supplement: Supplementary file 1 [file medicina-55-00114-s001.pdf]

medicina

Table S1. DMSO effects on viability of MCF-7 and MCF-10A.

Treatments	Cells	Viability (%)
Untreated cells	MCF-7	100±0.05
	MCF-10A	100±0.04
QT+DMSO	MCF-7	51.7±4.6*
	MCF-10A	99.7±0.23
DMSO	MCF-7	98.6±0.23
	MCF-10A	99.4±0.23

Values are expressed as mean ± SD (n=6). * $p < 0.01$, * indicates comparison to untreated cells.

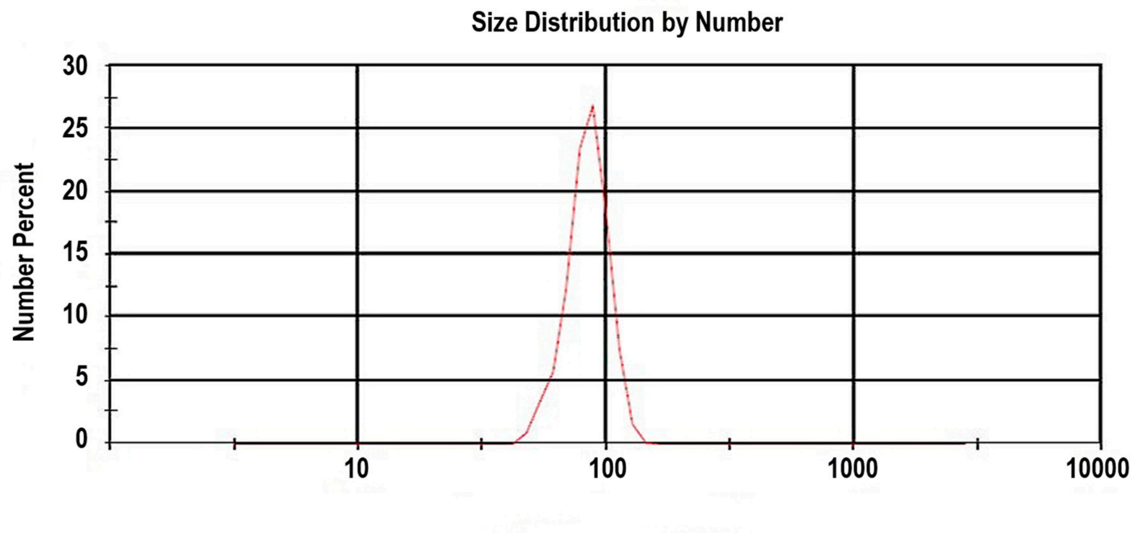

Figure S1. Size distribution of QT-loaded SLNs.

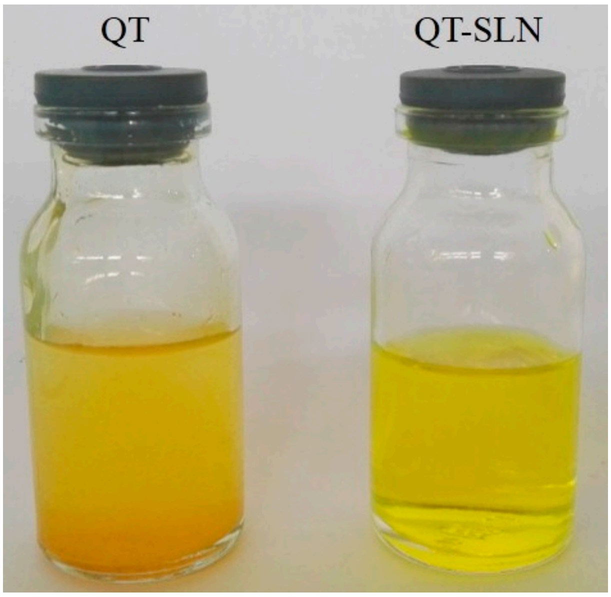

Figure S2. Free QT and QT-SLN in water.

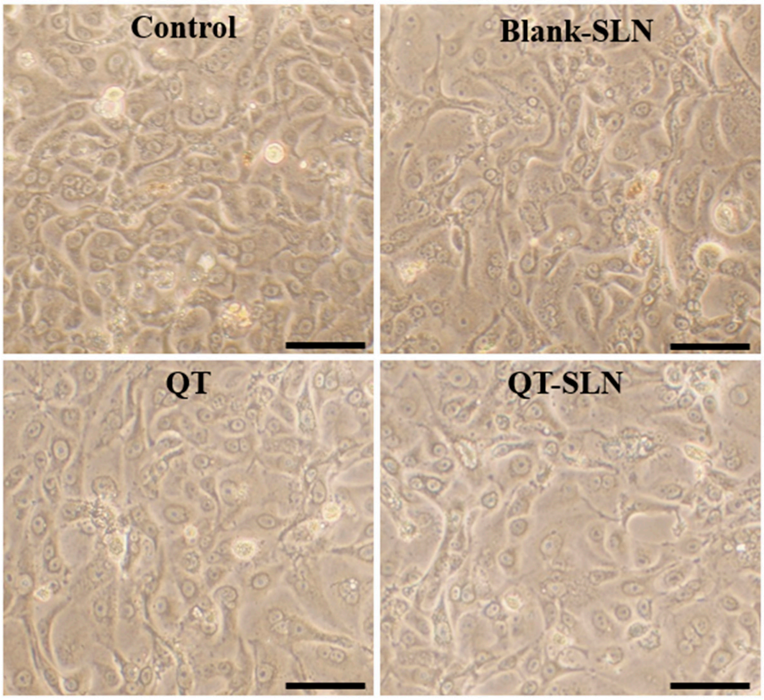

Figure S3. Morphology of MCF-10A cells in different groups. Scale bars: 100 μm.

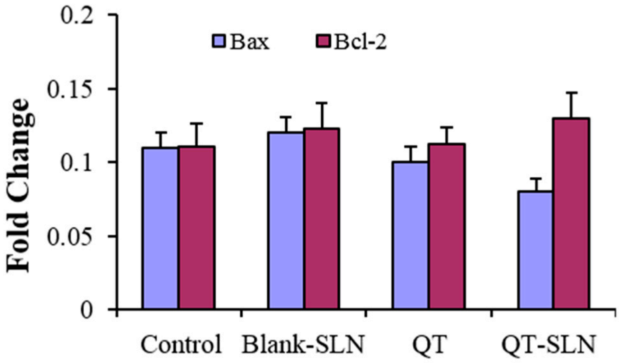

Figure S4. Gene expression for MCF-10A in different groups. Expression is normalized to average of housekeeping gene *(GAPDH).* Values are expressed as mean ± SD.
